# Survivable hypothermia or torpor in a wild-living rat: rare insights broaden our understanding of endothermic physiology

**DOI:** 10.1007/s00360-021-01416-3

**Published:** 2021-10-19

**Authors:** Julia Nowack, Christopher Turbill

**Affiliations:** 1 Hawkesbury Institute for the Environment and School of Science, Western Sydney University, Richmond, NSW Australia; 2 Present Address: School of Biological and Environmental Sciences, Liverpool John Moores University, Byrom Street, Liverpool, L3 3AF UK

**Keywords:** Heterothermy, Homeothermy, Native Australian rodent, Bush rat, Rewarming, Cooling

## Abstract

Maintaining a high and stable body temperature as observed in endothermic mammals and birds is energetically costly. Thus, it is not surprising that we discover more and more heterothermic species that can reduce their energetic needs during energetic bottlenecks through the use of torpor. However, not all heterothermic animals use torpor on a regular basis. Torpor may also be important to an individual’s probability of survival, and hence fitness, when used infrequently. We here report the observation of a single, ~ 5.5 h long hypothermic bout with a decrease in body temperature by 12 °C in the native Australian bush rat (*Rattus fuscipes*). Our data suggest that bush rats are able to rewarm from a body temperature of 24 °C, albeit with a rewarming rate lower than that expected on the basis of their body mass. Heterothermy, i.e. the ability to withstand and overcome periods of reduced body temperature, is assumed to be an evolutionarily ancestral (plesiomorphic) trait. We thus argue that such rare hypothermic events in species that otherwise appear to be strictly homeothermic could be heterothermic rudiments, i.e. a less derived form of torpor with limited capacity for rewarming. Importantly, observations of rare and extreme thermoregulatory responses by wild animals are more likely to be discovered with long-term data sets and may not only provide valuable insight about the physiological capability of a population, but can also help us to understand the constraints and evolutionary pathways of different phenologies.

## Introduction

As endotherms, mammals and birds are characterised by metabolically defending a near-constant body temperature (T_b_) by regulatory feedback mechanisms in the hypothalamus (Zhao et al. 2017). The maintenance of T_b_ within a narrow range of optimal temperatures has several advantages, such as sustained aerobic activity, independence from environmental conditions and optimisation of enzyme activity, but endothermy comes with a high energetic cost relative to ectothermic species (Nagy 2005). This energetic cost is particularly high for smaller species, and increases with decreasing ambient temperature (T_a_) below the animal’s thermal neutral zone (Bennett and Ruben 1979). Thus, given that food is often not continuously available, it is not surprising that some mammals and birds can lower their metabolically defended T_b_ in a controlled state known as torpor (Geiser 2004). During torpor, the metabolic rate of an animal is reduced in correspondence with the reduction in T_b_ and the animal is able to rapidly rewarm from its low T_b_ by endogenously fuelled heat production. This process is fundamentally different from unregulated hypothermia, during which T_b_ is equally reduced, but metabolic rate remains high as the animal is attempting to generate as much metabolic heat as possible to defend its T_b_ against a further decline. Torpor by so-called heterothermic endotherms, therefore, can provide a pronounced reduction of the animal’s daily energy expenditure (Geiser 2004). The resulting energy saving provides these species with greater flexibility in managing the combined risks of starvation and predation under variable environmental conditions (Turbill et al. 2011, 2019; Turbill and Stojanovski 2018). To date, we know more than 171 species of mammals and 43 bird species that are capable of using torpor (Ruf and Geiser 2015). Numbers of heterothermic species are steadily increasing, partly because ongoing technological advances in biologging equipment (Chmura et al. 2018) allow T_b_ to be recorded in a greater range of wild-living animals, which are more likely to exhibit torpor compared to captive animals (Geiser et al. 2000). Notable recent additions of species using torpor in the wild include a primate—the pygmy slow loris (Ruf et al. 2015), a passerine—the superb fairy-wren (Romano et al. 2019), and the discovery of multiday torpor in response to a flood event in golden spiny mice, a species that had been described previously as undergoing only short bouts of torpor for a few hours (Barak et al. 2018). While it is relatively easy to discover and describe torpor use in species that are using torpor on a regular basis, torpor could also be important to an individual’s probability of survival, and hence fitness, when used infrequently, such as in response to extreme weather events (Nowack et al. 2015), and these rare torpor bouts require long-term data sets of wild-living animals (see Nowack et al. (2020) for a recent list of species that are known to use torpor only rarely).

Australia’s native rodent species are one group of mammals that might be expected to use torpor, but for which we have very little data on T_b_, especially in wild-living animals. At least 40% of Australia’s native mammals are known to use torpor, yet although native rodents make up approximately 25% of Australian mammal species (Geiser and Körtner 2010), only one native species, the ash-grey mouse *Pseudomys albocinereus* has been shown to be capable of employing regulated torpor in the laboratory at T_a_ of 20 and 25 °C (Barker et al. 2012). Hypothermic bouts have also been recorded in the sandy mouse, *Pseudomys hermannsburgensis* (Tomlinson et al. 2007), but heterothermy in *P. hermannsburgensis* is assumed to represent hypothermia (Tomlinson et al. 2007), i.e. a non-controlled, non-reversible drop in T_b_. In addition, the house mouse, *Mus musculus*, an introduced species that lives in natural environments in Australia, shows daily torpor in the wild (Morton and Lee 1978) and in captivity in response to increased energy expenditure (Schubert et al. 2010) or increased perceived predation risk (Turbill and Stojanovski 2018), and a recent paper has revealed that juvenile mice use torpor during their development (Renninger et al. 2020). All other studies on thermoregulation of native Australian rodent species, such as the bush rat (*Rattus fuscipes*), so far suggest they are strictly homeothermic species that regulate their T_b_ within a narrow range (e.g. Glanville and Seebacher 2010). One reason for this might be a lack of field-based data. Bush rats are a particularly well-studied species, but all studies on bush rats have either been laboratory studies (Seebacher and Glanville 2010; Glanville et al. 2012) or short field studies lasting < 3 weeks (Glanville and Seebacher 2010), while longer term field data might be required to document the physiological breath of a species (Geiser et al. 2000, 2007). For example, studies comparing torpor use in sugar gliders under laboratory and field conditions have revealed differences in activity pattern as well as a difference in T_b_ regulation with a lower normothermic T_b_ and more frequent and deeper torpor bouts in the wild (Geiser et al. 2007).

We present long-term T_b_ data from free-ranging individuals of the native Australian bush rat (*Rattus fuscipes*), a species endemic to Australia, recorded during the austral autumn and early winter seasons. These data were collected during a broader study that aimed to test for within-individual correlations and among-individual differences in behavioural and physiological traits in wild rats. Among 1047 individual-days of T_b_, we recorded a single observation of a 12 °C decrease in T_b_ over 5.5 h during the resting phase for one individual. This paper focusses on this unique observation and discusses its implications for our understanding of endothermic thermoregulation.

## Methods

### Animals

We captured a total number of 30 bush rats in walk-in live traps (Elliott traps, type A) in the Blue Mountains, approximately 60 km WNW of Sydney, Australia (S 33.60994, E 150.63809) between March and May 2016. Traps were baited with rolled oats and peanut butter in the afternoon and checked in the early morning. After capture, rats were transported to Hawkesbury Campus of Western Sydney University, where they were fitted with a subcutaneous microchip for individual identification (PIT tag; Trovan ID100 Midichip) and surgically implanted with a temperature-sensitive data logger before being released (ibutton; DS1922L-F5#, resolution: 0.0625 °C, accuracy improved to 0.1 °C by calibration, logging interval: every 30 min; Maxim Integrated Products, Inc., Sunnyvale, California, USA; surgery details below). Temperature loggers were waxed in Elvax and calibrated before use in a water bath to the nearest of 0.1 °C (high precision NIST traceable digital thermometer; Control Company, Texas) from 5 to 40 °C. Loggers weighed < 3 g after waxing, which is < 5% of the body mass of adult bush rats (rats weighed between 59 and 137 g). After recovery from surgery (details below), rats were released at the exact capture location. We re-captured 14 of 30 implanted rats in June and July 2016 (recapture rate: 47%) and recorded a total of 1047 individual-days of T_b_ data between March and June 2016 (42–87 days per animal (Table 1), *N* = 14 animals (9 m, 5 f), average body mass: 92 ± 21 g).

**Table 1:** Overview of data recording

Animal	Release/recording start date	Recapture/recording end date	Number of days
1	14 March	02 June	80
2	14 March	01 June	79
3	14 March	01 June	79
4	20 March	14 June	87
5	27 March	12 June	77
6	27 March	12 June	77
7	27 March	12 June	77
8	27 March	12 June	77
9	04 April	19 June	76
10	04 April	19 June	76
11	12 April	28 June	77
12	12 April	28 June	77
13	22 April	29 June	66
14	17 May	29 June	42

The table indicates the recording period as well as the total number of recording days per animal

### Surgery details

Sterile transmitters were implanted intraperitoneally under general oxygen–isoflurane anaesthesia through a small (1–2 cm) abdominal incision. Pain relief was achieved by a single subcutaneous injection of a non-steroidal anti-inflammatory (carprofen). The skin and muscle fascia incisions were sutured separately using vicryl absorbable suture (Vicral Ethicon, Johnson & Johnson, Somerville, New Jersey, USA), and a topical anaesthetic (Xylocaine) and Leuko spray bandage plastic skin (BSN Medical, Clayton, Victoria, Australia) were applied. Post-surgery, animals were kept individually at 20 ± 2 °C and monitored closely. Animals were allowed to recover for up to 7 days before being released. All released animals appeared in good body condition and surgery wounds had healed well. Explantation of loggers from re-captured animals followed the same protocol as specified above.

### Ambient conditions

Ambient temperature was recorded within the trapping area via temperature loggers placed in the shade at a height of 1.2 m from the ground (ibutton, DS1922L-F5#, resolution: 0.0625 °C; Maxim Integrated Products, Inc., Sunnyvale, California, USA). Rainfall and solar radiation data for the whole study duration as well as minimum and maximum T_a_ for 6 days for which we were lacking ibutton data (14.03 to 19.03.2016) was sourced from the nearby weather station (~ 13 km away, Richmond RAAF, Australian Government Bureau of Meteorology; Pearson correlation coefficient was calculated to test the correlation between ibutton and weather station data and showed a high correlation for minimum (*R* = 0.92) as well as maximum T_a_ (*R* = 0.90)).

### Data analysis

Data analysis was conducted in R version 3.6.2 (R Development Core Team 2019). Data are presented as mean ± 1 standard deviation. *N* denotes the number of individuals, *n* the cumulative days of data recorded. T_b_ data were analysed by assigning each T_b_ datum to either nighttime (active-phase) or daytime (rest-phase) following Levesque et al. (2018). Plotting the frequency distribution of all data, we found a bi-modal distribution of T_b_ and calculated modal T_b_ of daytime (resting) and modal T_b_ of nighttime (activity). Sunset and sunrise data for the analysis and plots were calculated using the R package ‘maptools’ (Bivand and Lewin-Koh 2019), which is using the following website: https://www.esrl.noaa.gov/gmd/grad/solcalc/sunrise.html. Linear mixed effects models (“lme” in package ‘nlme’, Pinheiro et al. (2021)) followed by type 1 ANOVA were used to test the influence of minimum or maximum T_a_ on minimum, maximum T_b_ and the daily amplitude. All models accounted for repeated measures by including animal ID as random effect on the intercept. Normal distribution of residuals was checked visually and using Shapiro–Wilk tests and non-normally distributed data were boxcox transformed prior to carrying out the analysis. Pearson correlation coefficient was calculated to test the correlation between daily minimum and maximum T_a_. Rayleigh tests were used to determine whether circular data (timing of T_b_ above/below 38 °C) differed significantly from random distribution (“rayleigh.test” in package ‘circular’, Agostinelli and Lund (2017)).

## Results

### Ambient conditions

Mean daily T_a_ during the study period ranged from 5.8 to 22.6 °C, with an absolute nightly minimum of 0.6 °C at the end of May and an absolute daytime maximum of 34.6 °C at the end of March. Monthly average mean and maximum T_a_ decreased progressively from March to June, whereas average monthly minimum T_a_ was lowest in May (Table 1). Daily minimum and maximum T_a_ were strongly correlated (*R* = 0.99). Daylength decreased from just more than 12 h (12 h 6 min) in March to less than 10 h (9 h 54 min) in June. Daily amplitude in T_a_ ranged from 2 to 19 °C. Rainfall was low during March to May (4.2–12.8 mm per month) but increased in June (189.2 mm per month). Daily solar radiation was highest in March (monthly average: 16.3 MJ m^−2^) and decreased continuously until June (monthly average 8.8 MJ m^−2^), with an absolute minimum during the study of 2.2 MJ m^−2^ in early June.

### Variation in body temperature

**Fig. 1:**
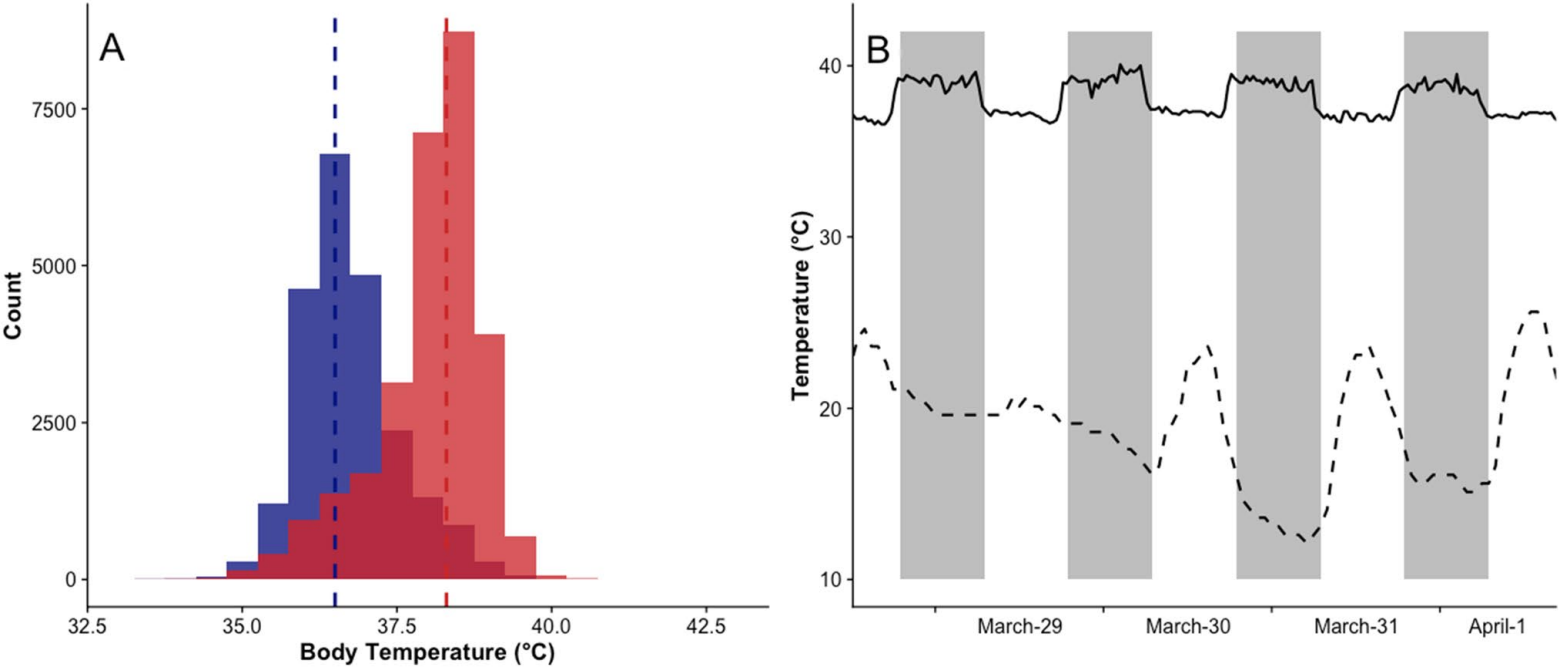
Body temperature pattern of bush rats **A** Body temperature distribution of bush rats (*N* = 14, *n* = 1047) over the total study duration (March-June). *Blue* bars represent daytime body temperature values, *red* bars nighttime values. **B** Exemplary body temperature trace over 4 days in March/April of a female bush rat. *Solid line:* body temperature, *dashed line*: ambient temperature, *shaded areas* illustrate nighttime.

Daily mean T_b_ of bush rats ranged between 37.4 and 37.6 °C during the study period (*N* = 14). T_b_ showed a clear bi-modal distribution with a daytime modal T_b_ of 36.5 °C and a nighttime modal T_b_ of 38.3 °C (Fig. 1A,B). Average daily maximum T_b_ was relatively stable and not influenced by minimum (*F*_1,1031_ = 3.4, *p* = 0.0636) or maximum T_a_ (*F*_1,1031_ = 1.6, *p* = 0.2032). Daily minimum T_b_ decreased from autumn to winter (Table 2) and was significantly influenced by minimum T_a_ (*F*_1,1031_ = 143.03, *p* < 0.001), i.e. rats had a lower T_b_ during colder days. Daily amplitude between minimum and maximum T_b_ ranged from 1.4 to 5.4 °C, with the exception of one occasion when an animal decreased its T_b_ by > 12 °C (see below), and daily T_b_ amplitude was significantly positively influenced by minimum T_a_ (*F*_1,1031_ = 126.5, *p* < 0.001, Table 2), as well as by daily amplitude in T_a_ (*F*_1, 1031_ = 20.5, *p* < 0.001).

**Table 2:** Monthly average body temperature and ambient temperature values, including number of sampling days (*n*)

Month	*n*	T_b_min (°C)	T_b_max (°C)	T_b_mean (°C)	T_b_amplitude (°C)	T_a_min (°C)	T_a_max (°C)
March	18	36.4 ± 0.5	39.1 ± 0.3	37.6 ± 0.4	2.8 ± 0.3	16.4 ± 3.0	27.6 ± 4.4
April	30	36.1 ± 0.3	39.1 ± 0.2	37.5 ± 0.2	3.0 ± 0.4	12.9 ± 2.0	21.9 ± 2.7
May	31	35.9 ± 0.4	39.1 ± 0.2	37.4 ± 0.3	3.2 ± 0.5	7.3 ± 2.8	19.2 ± 2.7
June	28	36.0 ± 0.5	39.1 ± 0.3	37.5 ± 0.3	3.1 ± 0.5	7.6 ± 3.1	15.0 ± 1.8

### Hypothermic bout

**Fig. 2:**
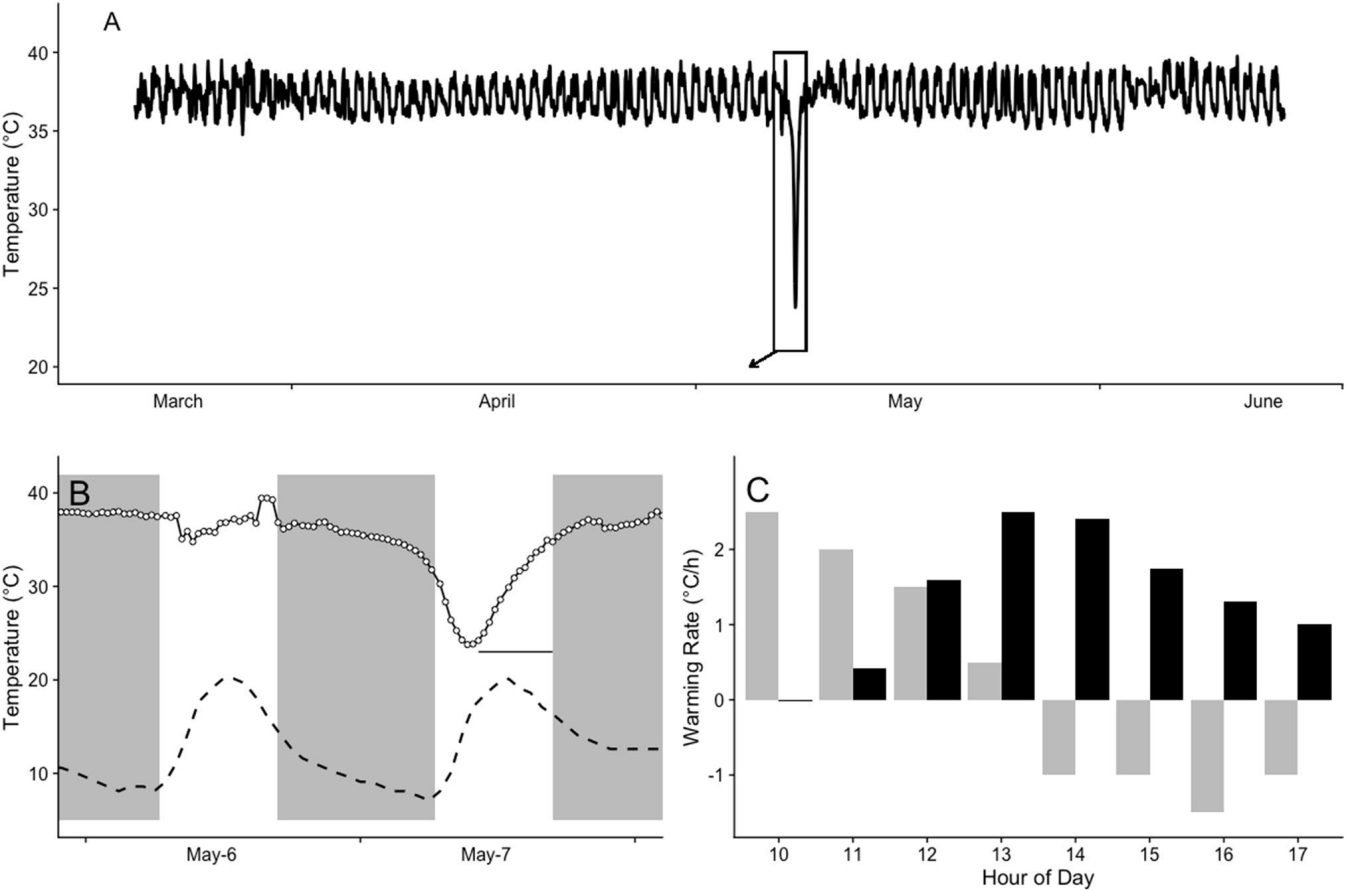
Hypothermic bout of female bush rat (*Rattus fuscipes*) **A** Body temperature recording from March to June **B** Body temperature trace of the days around the hypothermic bout; *solid line: body temperature, dashed line: ambient temperature, grey: night phase, horizontal line indicates the time period of warming body temperatures depicted in C.* **C** Warming rates of the animal (*black bars*) and the ambient temperature (*grey bars*). The animal started to rewarm at ~ 1100 h and a body temperature of ~ 35 °C was reached at around 1700 h, i.e. the start of the scotophase. Hourly values are calculated as the average between the previous hour and depicted hour

One subadult female (76 g at first capture) showed a large decrease in T_b_ beginning on the 7th of May (autumn; Fig. 2A, B), reaching a minimum daily T_b_ of 23.8 °C and T_b_ remaining < 30 °C for at least 5.5 h (0720 h to 1250 h; 30 min recording interval). On the day of the hypothermic bout, T_b_ of this individual peaked to above 39 °C at about 1.5 h before sunset and remained high for only 1.5 h before T_b_ was reduced to resting level (mean: 35.9 °C) and kept low until the early morning. On all other days in May, T_b_ only rose > 38 °C after sunset (mean 1753 h, timing different from random distribution: Rayleigh test, *r* = 0.9529, *p* < 0.001) and dropped back to < 38 °C only at a time just before sunrise (mean 0455 h, Rayleigh test: *r* = 0.908, *p* < 0.001). On the day of the hypothermic bout, T_b_ started to continuously decrease further at about 2.5 h before sunrise and reached a minimum at around 0850 h. Ambient temperature at this time was 15.2 °C (T_b_-T_a_ differential: 8.6 °C, Fig. 2).

**Fig. 3:**
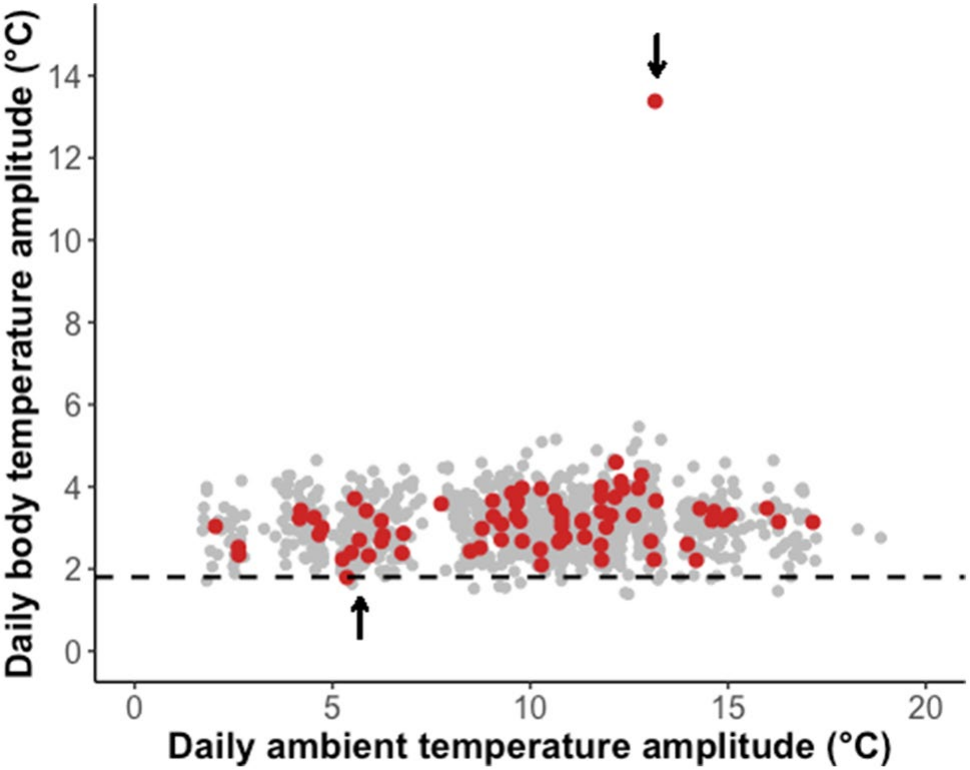
Daily body temperature amplitude versus daily ambient temperature amplitude. *Grey dots* represent all 14 bush rats, *red dots* represent the individual showing the hypothermic bout. The *dotted line* marks a body temperature amplitude of 1.8 °C for comparison purposes. The arrows mark day of the hypothermic bout (daily amplitude > 12 °C) and the day following the bout (1.8 °C)

Cooling rate of the animal during entry into the hypothermic bout varied from 0.5 to 3.9 °C h^−1^ measured over 10 min (i.e. < 0.01 to 0.07 °C min^−1^, Fig. 2). Maximum cooling rate was reached at a T_b_ of 30 °C and cooling rate slowed down again before reaching minimum T_b_. Minimum T_b_ of around 24 °C (23.8 to 24.3 °C) was maintained for about 1.5 h. Body temperature of the animal started to increase again in the early morning, reaching > 30 °C at around midday and back to a normal resting level T_b_ of ~ 35 °C in the afternoon (after ~ 6 h; Fig. 2A,B). Rewarming rate of the bush rat was fastest at a T_b_ below 30 °C and slowed down thereafter (Fig. 2C). Maximum rewarming rate of 2.75 °C h^−1^ (i.e. 0.05 °C min^−1^; Fig. 3) was reached at around 1200 h and was faster than warming of the environment (Fig. 2C). Ambient temperature reached a minimum of 8.1 °C at 0600 h and warmed thereafter, with a maximum warming rate occurring between 0800 and 0900 h. After 1000 h, T_a_ rose between 1.5 and 2.0 °C h^−1^ (~ 0.03 °C min^−1^) until 12 pm, then by 0.5 °C h^−1^ (< 0.01 °C min^−1^) until daytime maximum temperature of 21.7 °C was reached at 1 pm and T_a_ started to decrease again (Fig. 2C). The day of the hypothermic bout had no rainfall and a daily solar radiation of 13.7 MJ m^−2^.

The individual maintained a stable T_b_ on the day after the hypothermic bout with a daily amplitude of only 1.8 °C (Fig. 3). Body temperature did not drop below 34.8 °C on the other 76 days for which we recorded T_b_ in the field for this individual. The animal was re-captured in good body condition (body mass gain of + 12 g since last March) at the end of June.

## Discussion

Our data show that bush rats at our field site in Eastern Australia mostly kept their T_b_ within a narrow range, with a higher T_b_ during the nocturnal activity phase and a lower rest-phase T_b_. Minimum T_b_ of bush rats was lower on colder days and the amplitude between resting T_b_ and active T_b_ was largest during days with lower nightly minimum temperature. These data are largely in line with a previous field-based study of this species over a relatively short period of < 3 weeks per season that found a winter decrease in mean T_b_ and an increase in daily amplitude from 3.0 °C in summer to 3.6 °C winter (Glanville and Seebacher 2010). T_b_ levels varied slightly between both study populations and mean daily T_b_ was 0.6 °C to 0.8 °C higher in our study than in the previous study, potentially due to local habitat adaptations. However, in contrast to the strict homeothermic T_b_ regulation that the previous study has found (Glanville and Seebacher 2010), our data revealed a single hypothermic bout in one subadult individual in late autumn. This observation is the greatest daily reduction of T_b_ recorded under field conditions in the genus *Rattus* and provides evidence for either a survivable bout of profound hypothermia or a controlled bout of torpor in these endemic Australian bush rats.

### Does the heterothermic bout represent a torpor bout?

The observed decrease in T_b_ to a minimum of 23.8 °C was > 12 °C greater than the normal diurnal variation observed in this population and poses the question of whether we have observed a bout of torpor—as defined by a temporary, reversible and controlled reduction of metabolic rate and, at T_a_ less than T_b_, a corresponding reduction in T_b_ from normothermic values—or a bout of survivable hypothermia, which, in contrast to a torpor bout, involves high levels of metabolic heat production that nevertheless are insufficient to counter rates of heat loss, leading to an involuntary drop in T_b_ (Geiser et al. 2014).

Timing of entry and rewarming from low T_b_ was approximately corresponding with the early rest phase and thus similar to what has been reported for other daily heterotherms (Körtner and Geiser 2000). However, the early increase in T_b_ before sunset, i.e. during the normal resting time and the later maintenance of a resting level T_b_ during the active phase before the rather slow entry into the hypothermic bout are unusual for an otherwise nocturnal species. This almost certainly indicates that the individual did not exhibit activity and possibly did not forage for food during the night, and eventually was either not able to metabolically defend a high T_b_ and entered hypothermia or voluntarily dropped T_b_ to save energy during torpor.

Cooling and rewarming rates differ between regulated torpor and unregulated hypothermia: entry into hypothermia is slow at first, because of the high rate of metabolic heat production, and only becomes more rapid at lower T_b_, matching the exponential negative effect of temperature on enzyme activity (Geiser et al. 2014). Warming from hypothermia begins passively with rising T_a_, and hence usually slowly, and rewarming rate then increases with increasing T_b_ as metabolic activity is increased (Geiser et al. 2014). Based on this criterion, cooling in the bush rat resembles entry into hypothermia, because the rate increased only after the body had started to cool. Nevertheless, maximum cooling rate was comparable to cooling rates recorded during torpor entry in the much smaller ash-gray mice, *P. albocinereus,* the only native Australian rodent species for which torpor has been confirmed via T_b_ and metabolic rate data (Barker et al. 2012). Maximum cooling rate in ash-grey mice was 2.7 °C h^−1^, which is less than the maximum of 3.9 °C h^−1^ recorded for the individual bush rat, even though the bush rat was more than twice the body mass of *P. albocinerus* (15–40 g; Barker et al. 2012). However, cooling rates are difficult to compare between the studies, because it is also dependent on the T_a_-T_b_ differential, which was less for *P. albocinereus* at a T_a_ of 20 °C compared to that likely experienced by the bush rat.

The minimum T_b_ of the bush rat during torpor was maintained at ~ 24 °C for about 1.5 h, which was ~ 9 °C above external T_a_ at this time. This could suggest that T_b_ was maintained at a temporarily lowered set-point level such as found during regulated torpor (Heller and Colliver 1974), although it has to be considered that the microclimate surrounding the animal inside its nest might be buffered from the daily minimum T_a_ causing a lower T_a_-T_b_ differential. Further, the phase during which T_b_ was maintained at minimum level (i.e. the maintenance phase), might appear relatively short, but short maintenance phases during daily torpor are routinely observed in daily heterotherms, such as yellow footed antechinus, *Antechninus flavipes*, (Stawski et al. 2017; Reher et al. 2018) or the African lesser bushbaby, *Galago moholi* (Nowack et al. 2013b), and in some hibernating species, such as the Malagasy bat, *Macronycteris commersoni* (Reher et al. 2018).

The maximum rewarming rate of the bush rat was at 0.05 °C min^−1^ far less than expected for a similar-sized heterothermic species actively rewarming from torpor (0.3 °C min^−1^ for a 72 g animal) (Geiser and Baudinette 1990) and correspondingly, the duration of rewarming of 6 h was longer than would be expected. However, unlike rewarming from hypothermia (Geiser et al. 2014), the maximum rewarming rate of the bush rat was faster at the beginning and slowed down later during the rewarming process. Slower rates of rewarming than those expected on the basis of body mass have been reported for some species that evidently underwent regulated torpor, such as in the primate *G. moholi* (Nowack et al. 2013b), the native Australian rodent *P.albocinereus* (Barker et al. 2012), and the monotreme echidna *Tachyglossus aculeatus* (Nicol et al. 2009). Indeed, maximum rewarming rates in bush rats were about twice as fast as those recorded for *P. albocinereus* (2.75 °C h^−1^, or 0.05 °C min^−1^ for the bush rat vs. 1.5 °C h^−1^ or 0.025 °C min^−1^ for *P. albocinereus*) (Barker et al. 2012), even though the bush rat is larger than the ash-gray mouse and should thus rewarm slower.

The animal could also have passively rewarmed with T_a_, which increased in time with the initial period of rewarming of T_b_. If the animal has been in a buffered resting site, the increase in T_a_ might also have been delayed by a few hours. However, external T_a_ was ~ 9 °C lower than the animal’s T_b_ and the increase in T_a_ was slower than the maximum rewarming rate of the animal. Hence, passive rewarming of T_b_ by rising T_a_ alone could not have been entirely responsible for warming of the hypothermic animal. However, it is possible that an increasing T_a_ could have made active arousal easier by reducing the T_b_-T_a_ differential, especially during the earlier phase of rewarming. We think it highly unlikely the animal was exposed to direct solar radiation, because bush rats normally use underground burrows or ground nests under dense grass trees (Collins 1973; Frazer and Petit 2007). Basking in the sun should also have led to faster rewarming rates than those recorded. For example, dunnarts (*Sminthopsis* spp.) basking in sunlight can have a maximum passive arousal rates of up to 0.7 °C min^−1^ (Warnecke et al. 2008). Furthermore, two-step rewarming processes as observed in species that make use of passive rewarming normally start with an initial slow passive part and are followed by a faster active proportion after a certain T_b_ was reached (e.g. Schmid 2000). Rewarming in the bush rat followed the opposite pattern.

The question whether the recorded drop in T_b_ represents a controlled torpor bout or an unregulated bout of hypothermia unfortunately cannot be answered unequivocally based on the available data. Some aspects, such as the timing of entry and arousal and the successful rewarming suggest torpor, whereas others, such as the cooling process and the unusual low rewarming rate suggest it might instead have been a survivable bout of uncontrolled hypothermia.

### Implications of the hypothermic bout

Regardless of whether the bush rat was in a state of uncontrolled hypothermia or a controlled state of torpor, our field observation do show that bush rats are able to survive a drop in T_b_ to below 30 °C for ~ 5.5 h, and are able to rewarm, albeit slowly, from a T_b_ of ~ 24 °C with little if any reliance on external heat sources and without any apparent physiological damage. Small mammals have to cope with a high energy expenditure due to their small body size and high mass-specific rates of heat loss and they cannot survive for long without food intake while maintaining normothermic thermoregulation, especially when heat loss is increased at low T_a_ (Howard 1951). Allowing T_b_ to decrease can thus be beneficial and allow the animal to save energy, so long as the animal is able to rewarm again. Even lowering T_b_ by 1.2 °C leads to an energy saving of 6% of resting metabolic rate in a ~ 126 g marsupial mammal (i.e. sugar glider; Christian and Geiser 2007). The use of deep regulated torpor can even reduce energy expenditure to as little as to 1% of energy expenditure during normothermic conditions (data for a 18 g hibernating marsupial pygmy possum at Ta ≥ 5 °C; Geiser 1987), which can markedly increase an individual’s survival chances without access to food. Thus, the reduction in T_b_ observed in the bush rat would have reduced energy consumption, and such rare torpor bouts can be important for surviving exceptional circumstances during which foraging activity is impaired, such as unpredictable weather events (Nowack et al. 2017). However, the particular day was comparatively warm, with medium cloud cover (i.e. solar radiation) and no rainfall and thus the hypothermic bout observed for the bush rat cannot be explained by climatic conditions.

Unregulated hypothermia on the other hand can be lethal. When metabolic rate is not sufficient to maintain a stable elevated T_b_, T_b_ cools, and the high metabolic rate required for rewarming becomes impossible. Interestingly, even heterothermic species that can enter regulated torpor, such as the small marsupial antechinus (*Antechinus stuartii*), can become hypothermic when food deprived, i.e. they enter an unregulated state of hypothermia from which they cannot actively rewarm (Geiser 1988).

Laboratory studies report that at least some homeothermic rodents, i.e. species that are not known to undergo torpor, are able to survive periods of low T_b_ if they are subsequently warmed by external heat sources. Popovic (1960) found that adult laboratory rats were able to recover from hypothermia with a T_b_ as low as 15 °C, but only when hypothermia did not last more than 5.5 h (similar to our observation for the bush rat). When hypothermia lasted longer, rats were able to survive in a lethargic state at low T_b_ but did not survive being warmed up to normothermic values, which seemed to have been related to changes in blood pressure (Popovic 1960). Furthermore, animals exposed to severe hypothermia typically develop cardiac arrythmia and/or fibrillation (e.g. Biörck and Johansson 1955). The native Australian sandy mouse, *Pseudomys hermannsburgensis*, can survive extended hypothermia for up to 32.5 h with a minimum T_b_ of 17.3 °C at a T_a_ of 15 °C, when rewarmed passively (Tomlinson et al. 2007). In contrast, humans seem to be unable to survive deep hypothermia and generally maintain T_b_ in very strict boundaries (reviewed in Shi et al. 2021). Juvenile animals are usually better in surviving episodes of low T_b_. Hypothermic rat pups (*Rattus norvegicus)* can survive about 6 h of T_b_ below 30 °C under laboratory conditions, but need to be passively rewarmed (Geiser et al. 2014).

### How does this observation broaden our understanding of endotherm physiology?

So far, we know of at least four species that are physiologically able to undergo regulated torpor but seem to not use torpor on a regular basis. These include the sandy inland mouse, bushbabies, sugar glider (*Petaurus breviceps*; Christian and Geiser (2007)) and feathertail glider (*Acrobates pygmaeus;* Jones and Geiser (1992)) and these observations of rare torpor use seem thus to be restricted to non-Holarctic species (Nowack et al. 2020). Unpublished evidence also suggests that the native Australian swamp rat (*Rattus lutreolus*) can survive a decrease in T_b_ to 28 °C and seems able to rewarm spontaneously (C. Stawski, personal communication). Interestingly, at least two of the four species mentioned above also have low rewarming rates. As detailed above, rewarming rates for the bush rat are higher than those recorded for the sandy inland mouse, a species for which additional metabolic rate data have confirmed torpor use (Barker et al. 2012). Furthermore, the low rewarming rates from torpor observed in the African lesser bushbabies, *Galago moholi*, has led to the suggestion that bushbabies use torpor as a last resort strategy when energy reserves are low (Nowack et al. 2010, 2013b, a).

The finding of several mammal species that rarely use torpor with relatively low rewarming rates suggests these species can tolerate cold T_b_ but are not adapted to rewarm by massively increasing metabolic rate at low T_b_ like species that more regularly use torpor. While controversially discussed in the past (e.g. Geiser 2008), it is now assumed that ancestral mammals were largely heterothermic, i.e. had a high degree of thermoregulatory flexibility and survived periods of temporarily reduced T_b_ (Lovegrove 2012; Ruf and Geiser 2015), and that heterothermy may have played an important role in the evolution of endothermy (Wacker et al. 2017; Geiser et al. 2017). Torpor and hibernation are widespread across mammalian orders, yet not all recent mammal lineages are able to undergo torpor or to survive longer periods of hypothermia (Ruf and Geiser 2015), which means that the ability to express heterothermy must have been lost multiple times in the course of mammalian evolution, while other linages apparently evolved the more regulated form of torpor use. Thus, observations of rare torpor events in species that otherwise appear to be strictly homeothermic could be heterothermic rudiments, i.e. an intermediate form of torpor that involves survivable hypothermia with limited capacity for rewarming. Those data are only coming to light with long-term datasets of animals that capture their rare and extreme responses. Our data also emphasise the value of additionally recording metabolic rate (e.g. via heart rate; Currie et al. 2014) along with T_b_, which would allow to make assumptions about energy expenditure during the cooling period and rewarming process and can thus be helpful to differentiate between unregulated hypothermia and torpor use.

## Data Availability

Data are available on request.

## References

[CR1] Agostinelli C, Lund U (2017) R package ‘circular’: Circular Statistics (version 0.4–93). https://r-forge.r-project.org/projects/circular/

[CR2] Barak O, Geiser F, Kronfeld-Schor N (2018). Flood-induced multiday torpor in golden spiny mice (*Acomys russatus*). Aust J Zool.

[CR3] Barker JM, Cooper CE, Withers PC, Cruz-Neto AP (2012). Thermoregulation by an Australian murine rodent, the ash-grey mouse (*Pseudomys albocinereus*). Comp Biochem Physiol A Mol Integr Physiol.

[CR4] Bennett A, Ruben J (1979). Endothermy and activity in vertebrates. Science.

[CR5] Biörck G, Johansson B (1955). Comparative studies on temperature effects upon the electrocardiogram in some vertebrates. Acta Physiol Scand.

[CR6] Bivand R, Lewin-Koh N (2019) Maptools: tools for handling spatial objects. R package version 0.9–9.

[CR7] Chmura HE, Glass TW, Williams CT (2018). Biologging physiological and ecological responses to climatic variation: new tools for the climate change era. Front Ecol Evol.

[CR8] Christian N, Geiser F (2007). To use or not to use torpor? Activity and body temperature as predictors. Naturwissenschaften.

[CR9] Collins BG (1973). The ecological significance of thermoregulatory responses to heat stress shown by two populations of an Australian murid, *Rattus fuscipes*. Comp Biochem Physiol A Physiol.

[CR10] Currie SE, Körtner G, Geiser F (2014). Heart rate as a predictor of metabolic rate in heterothermic bats. J Exp Biol.

[CR11] Frazer DS, Petit S (2007). Use of *Xanthorrhoea semiplana* (grass-trees) for refuge by *Rattus fuscipes* (Southern bush rat). Wildl Res.

[CR12] Geiser F (1987). Hibernation and daily torpor in two pygmy-possums (*Cercartetus *spp., Marsupialia). Physiol Zool.

[CR13] Geiser F (1988). Daily torpor and thermoregulation in antechinus (Marsupialia): influence of body mass, season, development, reproduction, and sex. Oecologia.

[CR14] Geiser F (2004). Metabolic rate and body temperature reduction during hibernation and daily torpor. Annu Rev Physiol.

[CR15] Geiser F (2008). Ontogeny and phylogeny of endothermy and torpor in mammals and birds. Comp Biochem Physiol A Mol Integr Physiol.

[CR16] Geiser F, Baudinette RV (1990). The relationship between body mass and rate of rewarming from hibernation and daily torpor in mammals. J Exp Biol.

[CR17] Geiser F, Körtner G (2010). Hibernation and daily torpor in Australian mammals. Aust Zool.

[CR18] Geiser F, Holloway JC, Körtner G, Maddocks TA, Turbill C, Brigham RM, Heldmaier G, Klingenspor M (2000). Do patterns of torpor differ between free-ranging and captive mammals and birds?. Life in the Cold.

[CR19] Geiser F, Holloway J, Körtner G (2007). Thermal biology, torpor and behaviour in sugar gliders: a laboratory-field comparison. J Comp Physiol B.

[CR20] Geiser F, Currie SE, O'Shea KA, Hiebert SM (2014). Torpor and hypothermia: reversed hysteresis of metabolic rate and body temperature. Am J Physiol Regul Integr Comp Physiol.

[CR21] Geiser F, Stawski C, Wacker CB, Nowack J (2017). Phoenix from the ashes: fire, torpor, and the evolution of mammalian endothermy. Front Physiol.

[CR22] Glanville EJ, Seebacher F (2010). Plasticity in body temperature and metabolic capacity sustains winter activity in a small endotherm (*Rattus fuscipes*). Comp Biochem Physiol A Mol Integr Physiol.

[CR23] Glanville EJ, Murray SA, Seebacher F (2012). Thermal adaptation in endotherms: climate and phylogeny interact to determine population-level responses in a wild rat. Funct Ecol.

[CR24] Heller HC, Colliver GW (1974). CNS regulation of body temperature during hibernation. Am J Physiol.

[CR25] Howard WE (1951). Relation between low temperature and available food to survival of small rodents. J Mammal.

[CR26] Jones CJ, Geiser F (1992). Prolonged and daily torpor in the feathertail glider, *Acrobates pygmaeus* (Marsupialia: Acrobatidae). J Zool.

[CR27] Körtner G, Geiser F (2000). The temporal organization of daily torpor and hibernation: circadian and circannual rhythms. Chronobiol Int.

[CR28] Levesque DL, Tuen AA, Lovegrove BG (2018). Staying hot to fight the heat-high body temperatures accompany a diurnal endothermic lifestyle in the tropics. J Comp Physiol B.

[CR29] Lovegrove BG (2012). The evolution of endothermy in Cenozoic mammals: a plesiomorphic-apomorphic continuum. Biol Rev.

[CR30] Morton SR, Lee AK (1978). Thermoregulation and metabolism in *Planigale maculata* (Marsupialia: Dasyuridae). J Therm Biol.

[CR31] Nagy KA (2005). Field metabolic rate and body size. J Exp Biol.

[CR32] Nicol SC, Andersen NA, Arnold W, Ruf T (2009). Rewarming rates of two large hibernators: comparison of a monotreme and a eutherian. J Therm Biol.

[CR33] Nowack J, Mzilikazi N, Dausmann KH (2010). Torpor on demand: heterothermy in the non-lemur primate *Galago moholi*. PLoS ONE.

[CR34] Nowack J, Dausmann KH, Mzilikazi N (2013). Nonshivering thermogenesis in the African lesser bushbaby, *Galago moholi*. J Exp Biol.

[CR35] Nowack J, Mzilikazi N, Dausmann KH (2013). Torpor as an emergency solution in *Galago moholi*: heterothermy is triggered by different constraints. J Comp Physiol B.

[CR36] Nowack J, Rojas AD, Körtner G, Geiser F (2015). Snoozing through the storm: torpor use during a natural disaster. Sci Rep.

[CR37] Nowack J, Stawski C, Geiser F (2017). More functions of torpor and their roles in a changing world. J Comp Physiol B.

[CR38] Nowack J, Levesque DL, Reher S, Dausmann KH (2020). Variable climates lead to varying phenotypes: “weird” mammalian torpor and lessons from non-holarctic species. Front Ecol Evol.

[CR39] Pinheiro J, Bates D, DebRoy S, Sarkar D, R Core Team (2021) nlme: linear and nonlinear mixed effects models. R package version 3.1-153. https://CRAN.R-project.org/package=nlme

[CR40] Popovic V (1960). Physiological characteristics of rats and ground squirrels during prolonged lethargic hypothermia. Am J Physiol.

[CR41] R Development Core Team (2019). R: a language and environment for statistical computing.

[CR42] Reher S, Ehlers J, Rabarison H, Dausmann KH (2018). Short and hyperthermic torpor responses in the Malagasy bat Macronycteris commersoni reveal a broader hypometabolic scope in heterotherms. J Comp Physiol B.

[CR43] Renninger M, Sprau L, Geiser F (2020). White mouse pups can use torpor for energy conservation. J Comp Physiol B.

[CR44] Romano AB, Hunt A, Welbergen JA, Turbill C (2019). Nocturnal torpor by superb fairy-wrens: a key mechanism for reducing winter daily energy expenditure. Biol Lett.

[CR45] Ruf T, Geiser F (2015). Daily torpor and hibernation in birds and mammals. Biol Rev.

[CR46] Ruf T, Streicher U, Stalder GL, Nadler T, Walzer C (2015). Hibernation in the pygmy slow loris (*Nycticebus pygmaeus*): multiday torpor in primates is not restricted to Madagascar. Sci Rep.

[CR47] Schmid J (2000). Daily torpor in the gray mouse lemur (*Microcebus murinus*) in Madagascar: energetic consequences and biological significance. Oecologica.

[CR48] Schubert KA, Boerema AS, Vaanholt LM, de Boer SF, Strijkstra AM, Daan S (2010). Daily torpor in mice: high foraging costs trigger energy-saving hypothermia. Biol Lett.

[CR49] Seebacher F, Glanville EJ (2010). Low levels of physical activity increase metabolic responsiveness to cold in a rat (*Rattus fuscipes*). PLoS ONE.

[CR50] Shi Z, Qin M, Huang L, Xu T, Chen Y, Hu Q, Peng S, Peng Z, Qu L-N, Chen S-G, Tuo Q-H, Liao D-F, Wang X-P, Wu R-R, Yuan T-F, Li Y-H, Liu X-M (2021). Human torpor: translating insights from nature into manned deep space expedition. Biol Rev.

[CR51] Stawski C, Nowack J, Körtner G, Geiser F (2017). A new cue for torpor induction: charcoal, ash and smoke. J Exp Biol.

[CR52] Tomlinson S, Withers PC, Cooper C (2007). Hypothermia versus torpor in response to cold stress in the native Australian mouse *Pseudomys hermannsburgensis* and the introduced house mouse *Mus musculus*. Comp Biochem Physiol A Mol Integr Physiol.

[CR53] Turbill C, Stojanovski L (2018). Torpor reduces predation risk by compensating for the energetic cost of antipredator foraging behaviours. Proc Royal Soc B Biol Sci.

[CR54] Turbill C, Bieber C, Ruf T (2011). Hibernation is associated with increased survival and the evolution of slow life histories among mammals. Proc Royal Soc B Biol Sci.

[CR55] Turbill C, McAllan BM, Prior S (2019). Thermal energetics and behaviour of a small, insectivorous marsupial in response to the interacting risks of starvation and predation. Oecologia.

[CR56] Wacker CB, McAllan BM, Körtner G, Geiser F (2017). The role of basking in the development of endothermy and torpor in a marsupial. J Comp Physiol B.

[CR57] Warnecke L, Turner J, Geiser F (2008). Torpor and basking in a small arid zone marsupial. Naturwissenschaften.

[CR58] Zhao ZD, Yang WZ, Gao C, Fu X, Zhang W, Zhou Q, Chen W, Ni X, Lin JK, Yang J, Xu XH, Shen WL (2017). A hypothalamic circuit that controls body temperature. Proc Natl Acad Sci USA.

